# Prevalence of *Toxoplasma gondii* IgG Antibodies and Associated Risk Factors in Psychiatric Patients from Western Romania: A Cross-Sectional Study

**DOI:** 10.3390/microorganisms12010172

**Published:** 2024-01-15

**Authors:** Sebastian Grada, Alin Gabriel Mihu, Daniela Adriana Oatis, Monica Susan, Maria Alina Lupu, Tudor Rares Olariu

**Affiliations:** 1 Discipline of Parasitology, Department of Infectious Disease, Victor Babes University of Medicine and Pharmacy, 300041 Timisoara, Romania; seba.grada@gmail.com (S.G.); lupu.alina@umft.ro (M.A.L.); 2 Center for Diagnosis and Study of Parasitic Diseases, Victor Babes University of Medicine and Pharmacy, 300041 Timisoara, Romania; 3 Department of Biology and Life Sciences, Vasile Goldis Western University, 310300 Arad, Romania; 4 Patogen Preventia, 300124 Timisoara, Romania; 5 Department of Infectious Disease, Faculty of Medicine, Vasile Goldis Western University, 310300 Arad, Romania; danielaoatis@gmail.com; 6 Centre for Preventive Medicine, Department of Internal Medicine, “Victor Babes” University of Medicine and Pharmacy, 300041 Timisoara, Romania; monisusan@yahoo.com; 7 Clinical Laboratory, Institute of Cardiovascular Diseases, 300310 Timisoara, Romania; 8 Clinical Laboratory, Municipal Clinical Emergency Teaching Hospital, 300254 Timisoara, Romania

**Keywords:** Western Romania, psychiatric patients, serology, risk factors, *Toxoplasma gondii*

## Abstract

Infection with the coccidian parasite *Toxoplasma gondii* was associated with an increased risk of several mental disorders. We conducted a case–control study of 464 consecutive psychiatric patients and assessed the prevalence of IgG antibodies against *T. gondii* and the potential risk factors associated with infection. *T. gondii*-specific antibodies were determined using a chemiluminescence assay. A questionnaire was utilized to assess the potential correlation between risk factors and *Toxoplasma gondii* seropositivity. IgG antibodies were found in 325 (70.04%) of the patients. We observed a higher likelihood of positive IgG antibodies against *Toxoplasma gondii* in older individuals, patients residing in rural areas, and females. We also noted associations between *Toxoplasma gondii* infection and certain risk factors, like activities that involve contact with soil, low-income levels, and limited educational attainment. Our findings indicate a high prevalence of *T. gondii* infection among psychiatric patients from Western Romania and provide new information regarding the potential risk factors associated with *T. gondii* in this population group. This study may serve as a foundation for future research and the development of preventive strategies.

## 1. Introduction

*Toxoplasma gondii* is a coccidian parasite that infects warm-blooded animals and causes a zoonotic parasitic disease called toxoplasmosis [1,2]. *T. gondii* infects approximately one-third of the world’s population [1]. Humans are considered accidental hosts of this parasite and can be infected in a variety of ways. Contact with this parasite may occur through the ingestion of tissue cysts in undercooked or contaminated meat (most often pork, lamb, goat, or wild meat), consumption of unwashed fruits or vegetables, contaminated water, and contact with contaminated soil or cat litter [3,4]. A different route of transmission for *T. gondii* is through organ transplantation or blood transfusions from an infected donor to an uninfected recipient [5].

Acute toxoplasmosis is usually asymptomatic in immunocompetent individuals [6]. However, in pregnant women, acute toxoplasmosis has been linked to significant damage to the foetus with drastic consequences, such as ocular and neurological disorders (chorioretinitis, intracranial calcifications, microcephaly or hydrocephalus, and intellectual disabilities) [7,8,9].

In immunosuppressed individuals, acute toxoplasmosis is most often the result of reactivation of a latent infection presenting neurological signs such as headache, disorientation, drowsiness, hemiparesis, reflex changes, and convulsions [10].

Chronic toxoplasmosis is, in the vast majority of cases, asymptomatic [6,11,12]. However, studies performed in previous years indicated that patients with detectable T. *gondii* IgG antibodies are more likely to be at risk for different psychiatric disorders [13,14,15]. Latent toxoplasmosis has been associated with tissue cysts of *T. gondii* in the brain tissue [16,17]. Previous studies showed an association between *T. gondii* antibody seropositivity and the presence of schizophrenia [18,19,20], bipolar disease [21,22], suicide attempts [23], self-directed violence [24], depression [25], and alcohol abuse [26].

Acute and chronic toxoplasmosis may be diagnosed using serological tests. A combination of tests for the detection of IgM, IgG, IgA-specific antibodies, and IgG avidity are useful to differentiate between acute and chronic infection. The presence of detectable IgG antibodies against *T. gondii* accompanied by undetectable IgM and IgA and high avidity of IgG antibodies is regarded as a chronic infection. Positive IgM, IgA against *T. gondii*, and low IgG avidity are considered indicative of acute toxoplasmosis [2,12,27].

Several investigators suggested that *T. gondii* could play a role in the aetiology of psychiatric disorders through the generated immune response involving the production of interferon-gamma [1,28]. This cytokine induces astrocytes to produce an enzyme called indoleamine-2, 3-dioxygenase, which degrades tryptophan (used for *T. gondii* replication) and induces the production of neurotoxic metabolites. These metabolites also influence the balance of neurotransmitters. *T. gondii* can also directly enhance dopamine activity, a critical process in psychiatric disorder pathogenesis [1,29,30].

Chronic toxoplasmosis may also influence the glutamate and gamma-aminobutyric acid (GABA) neurotransmitter system. Fuks et al. demonstrated that *T. gondii* may enter the brains of mice and trigger the production of GABA [31]. The effects of *T. gondii* infection may extend beyond GABA and dopamine, further causing alterations in neurotransmitters such as glutamate, serotonin, and norepinephrine in mouse models [32]. *T. gondii* may also cause alterations in the tryptophan metabolism pathway [33]. Depletion of tryptophan may result in a decrease in serotonin levels and potentially may contribute to an increase in suicide attempt rates and even suicides in infected individuals [34].

Limited epidemiological data on the relationship between *T. gondii* infection and psychiatric diseases were published in Romania. The current literature on this subject did not evaluate the possible contamination routes and risk factors that may be associated with infection in psychiatric patients [35,36]. Therefore, we performed a cross-sectional study in a population of psychiatric patients residing in Arad County, Western Romania, to estimate the prevalence of *T. gondii* IgG antibodies and to further identify the potential risk factors that may lead to possible infection with this protozoan parasite.

## 2. Subjects and Methods

### 2.1. Study Population

The study group included 464 consecutive psychiatric adult patients who were admitted to the Psychiatric Clinic of County Emergency Hospital of Arad in Western Romania between 1 July 2018 and 31 July 2019. Hospitalized patients diagnosed with a psychiatric disorder at admission were included in this study. Patients who were unable to complete the questionnaire due to the nature of their illness and who did not have a primary caregiver (who was able to help them complete the questionnaire) were excluded from this study. Clinical diagnoses were established in accordance with the Diagnostic and Statistical Manual of Mental Disorders, Fifth Edition (DSM-5). Mental illnesses were further classified using the International Classification of Disease, 10th Revision (ICD-10) codes. Patients were grouped by age into the following categories: 19–29 years, 30–39 years, 40–49 years, 50–59 years, 60–69 years, and 70+ years.

### 2.2. Serologic Tests

Blood samples were collected using standard venipuncture methods into Serum Separation Gel and Clot Activator Vacuum Tubes (Becton Dickinson, Vaud, Switzerland). The collected samples were centrifuged 2000× *g* for 10 min, 10 to 30 min after collection. The obtained sera were moved into Sterile Centrifuge Eppendorf Tubes and stored at −70 °C until the *T. gondii* IgG antibodies were analysed.

*T. gondii* IgG antibodies were determined using chemiluminescence on an Immulite 2000 analyser (Siemens Healthcare Diagnostics, Malvern, PA, USA) in accordance with manufacturer’s instructions and internal laboratory standards. Immulite 2000 *T. gondii* IgG is an all-2-step, solid-phase enzyme-enhanced immunoassay [37]. Values below 6.5 IU/mL were considered negative, values between 6.5 IU/mL and 8 IU/mL were considered equivocal, and values above 8 IU/mL were considered positive. For the purpose of this study, equivocal results were considered negative.

### 2.3. Questionnaires

The participants voluntarily filled out a self-administered questionnaire. When the patients were unable to fulfil the request due to the nature of their illness, the primary caregiver was tasked to complete the questionnaire. The whole process was conducted by specialized nurses under the strict supervision of the principal investigators. The data collection tool consisted of two parts. The first part of the questionnaire included the basic demographic characteristics, such as age, sex, and area of residence. The second part included risk factors, such as contact with soil (e.g., gardening or farming); contact with cats (defined as daily interaction with a cat); contact with dogs (defined as daily interaction with a dog); income level (defined as high (RON > 2500 or 500 EUR/month) and low (RON ≤ 2500 or 500 EUR/month)); education attainment (higher education (>12 grades) and lower education (≤12 grades)); hand sanitizing prior to food preparation; consumption of treated public water, meat and undercooked meat consumption; unwashed horticultural products (fruits and vegetables); consumption of alcohol (beer, wine, brandy, or vodka); and residents in the same household (≤2 residents and >2 residents in the same household).

### 2.4. Statistical Analysis

Data were collected using Microsoft Excel, version 2011 (Microsoft Corp., Redmond, WA, USA). Statistical analyses were performed with the Epi Info statistical package 3.3.2 (Centres for Disease Control and Prevention, Atlanta, GA, USA). Categorical data were presented as percentages. For comparison tests between groups, we used Mantel–Haenszel chi-square and two-tailed Fisher’s exact tests. The *p* values for all hypothesis tests were two-sided, and statistical significance was set at *p* < 0.05.

### 2.5. Informed Consent and Ethics

This study was approved by the Ethics Committee of Emergency County Hospital of Arad, Romania (No. 8051 from 16 March 2018) and by Victor Babes University Ethics Committee, Timisoara, Romania (No. 05/16 January 2018). All participants signed a written informed consent.

## 3. Results

Of the 464 psychiatric patients enrolled in this study aged between 19 and 97 years (mean age 52.08 ± 12,89 years), 258 (55.5%) were residing in rural areas, and 245 (52.7%) were female. *T. gondii* IgG antibodies were found in 325 of 464 (70.04%) study participants and the seroprevalence tended to increase with age (Table 1).

A significant difference between psychiatric patients with detectable *T. gondii* antibodies and patients with undetectable antibodies was found when psychiatric patients aged between 40 and 49 years (OR: 4.26, 95%CI: 1.67–10.89, *p* = 0.003), 50 to 59 years (OR: 4.69, 95% CI: 1.91–11.49, *p* = 0.007), 60 to 69 years (OR: 4.26, 95%CI: 1.67–10.89, *p* = 0.003) and patients aged 70+ years (OR: 8.67, 95%CI: 2.45–30.69, *p* < 0.001) were compared to patients aged 19 to 29 years (Table 1).

Our results indicate a significant difference between psychiatric patients with detectable *T. gondii* antibodies and patients with undetectable antibodies residing in rural areas when compared to study participants residing in urban areas (OR: 1.54, 95%CI: 1.03–2.28, *p* = 0.04) (Table 1). A difference in the seroprevalence of *T. gondii* IgG antibodies was also found when female psychiatric patients were compared to male patients (OR: 1.53, 95%CI: 1.03–2.28, *p* = 0.04) (Table 1).

Among specific psychiatric disorders, intellectual disabilities presented the highest seroprevalence of *T. gondii* IgG antibodies (85.71%), followed by delusional disorders (85.19%), dementia (82.76%), alcohol abuse (78.57%), other personality and behavioural disorders (70.83%), schizophrenia (70.75%), and depression (70.43%). Other disorders with notable seroprevalence rates included somatic symptom disorders, bipolar disorder, mood disorders, impulse control disorders, and adjustment disorders (Table 2).

Our results indicate a significantly higher prevalence of *T. gondii* IgG antibodies in psychiatric patients who reported engaging in activities involving contact with the soil when compared to patients who reported not engaging in such activities (OR: 1.56, 95% CI: 1.04–2.32, *p* = 0.03).

A relevant decrease in seroprevalence of *T. gondii* IgG antibodies was observed in psychiatric patients with high income when compared to those with low income (OR: 0.62, 95% CI: 0.41–0.93, *p* = 0.02).

Further analysis of the data confirmed a reduction in the seroprevalence of *T. gondii* IgG antibodies when psychiatric patients with higher education were compared to patients with lower education (OR: 0.58, 95% CI: 0.39–0.87, *p* = 0.009).

Other risk factors, such as contact with cats, contact with dogs, hand sanitizing prior to food preparation, consumption of publicly treated water, consumption of meat and uncooked meat, unwashed horticultural products, consumption of alcohol, and the number of residents in the same household did not show a notable association with *T. gondii* IgG seropositivity (Table 3).

## 4. Discussion

Recent studies highlighted the potential correlation between chronic *T. gondii* infection and psychiatric disorders [38,39]. This association may be underpinned by various mechanisms through which *T. gondii* exerts its influence on the central nervous system. Numerous studies have indicated that chronic *T. gondii* infection holds the potential to induce alterations in human behaviour, contributing to the development of psychiatric disorders [30]. These behavioural changes associated with *T. gondii* infection are believed to stem from the direct impact of the parasite, which can lead to nerve damage. Importantly, the cytokine IFN-γ, primarily responsible for immunological defence against *T. gondii*, plays a pivotal role in all infected tissues, including the central nervous system [40,41,42]. Furthermore, *T. gondii* is a neurotropic organism employing intricate mechanisms to access the brain, where it forms cysts within glial cells, neurons, and astrocytes. This process results in the development of numerous foci characterized by enlarging necrosis and the formation of microglia nodules. Notably, *T. gondii* possesses the capability to integrate its genetic material into the host’s deoxyribonucleic acid (DNA), potentially influencing brain function or modulating the growth of neuronal cells in vitro [43,44]. The interaction between *T. gondii* and the central nervous system involves a complex interplay of neuroinflammatory responses and neurochemical alterations, providing additional insights into the intricate relationship between this parasite and the pathogenesis of psychiatric conditions [45].

In the present study, we have identified a *T. gondii* IgG antibody prevalence rate of 70.04% among psychiatric patients, similar to the rates reported in 2022 in Arad County (67.86%) [36], but higher than the prevalence estimated in psychiatric patients from Timis County (54.7%) in 2017 from Western Romania [46]. Our reported *T. gondii* seroprevalence is also higher than the seroprevalence of 13.3% reported by Chen et al. [15] in China, 21.7% reported by Abd El-Aal et al. [47] in Egypt, 30.7% reported by James et al. [48] in Nigeria, 50.3% reported by Elsaid et al. [49] in Libya—similar to 70% reported by Kezai et al. [50] in psychiatric patients with schizophrenia from Algeria, but lower than a seroprevalence of 74.8% Esshili et al. [51] reported in psychiatric patients with schizophrenia and 76.9% Hamdani et al. [52] reported in bipolar patients from France. Notably, the seroprevalence in the present study significantly exceeded the global estimate of 38.27% and the reported European prevalence of 57% in psychiatric patients, as recently identified in a comprehensive meta-analysis encompassing 1250 studies from 18 countries [39].

Psychiatric patients from rural areas had a higher rate of positive *T. gondii* antibodies when compared with psychiatric patients residing in urban areas. These findings are in contrast with other studies conducted on psychiatric patients, who found no association between *T. gondii* seropositivity and area of residence [53,54,55]. A possible explanation is that activities carried out in rural areas may expose individuals to soil or water contaminated with oocysts [56], and these activities may vary from region to region. Activities that include gardening, farming, and handling animals are factors that may contribute to a higher prevalence of *T. gondii* IgG antibodies in psychiatric patients in rural areas [57].

Female psychiatric patients had a higher chance of contacting *T. gondii* in Western Romania. Our findings are in accordance with a previous report from psychiatric patients [36] from the same region.

Psychiatric patients who engaged in activities that involved contact with the soil had higher rates of *T. gondii* infection. These results are in line with those obtained by Achaw et al. [54] in psychiatric patients from Gondar, Northern Ethiopia, Cong et al. [58] in Eastern China, and Zaki et al. [59] in Jazan Province, Saudi Arabia. Human infection has historically been associated with significant risk factors linked to exposure to soil [60]. In natural settings, it was observed that *T. gondii* oocysts were infectious in the soil for up to 18 months [61].

The results of the present study did not find an association between *T. gondii* IgG antibody positivity and ownership of cats. These results are consistent with results obtained by Elsaid et al. [49] in Libya. A possible explanation is that cats only pose a risk of infection when they are actively shedding oocysts [3], which lasts a short period of time (a maximum of 21 days) [3,62].

In the current research, contact with dogs did not have significant effects on *T. gondii* seropositivity. Our findings are in agreement with the results of Flegr et al. [63].

The present investigation revealed that psychiatric patients with a low income tend to have a higher prevalence of *T gondii* antibodies when compared to the ones with a high income, similar to results published by Gale et al. [64] and Owusu-Dommey et al. [65] in the USA, as well as Mareze et al. [66] in Brazil. Low-income levels are linked with substandard living conditions and deficient hygiene practices, thereby elevating the susceptibility to contracting this protozoan parasite [66,67].

Another potential risk factor for *T. gondii* was the education level of our study participants. *T. gondii* seropositivity was higher in patients with a lower education level when compared to those with a higher education level, as previously observed by Șirin et al. [68] in bipolar patients, Gale et al. [64] in the general population, and Bigna et al. [67] in pregnant females.

The current results point out that hand washing does not play a crucial role in contacting the parasite. A similar finding was reported by Achaw et al. [54], who found no association between hand washing and *T. gondii* seroprevalence.

No evidence of a connection between the consumption of tap water and *T. gondii* infection among psychiatric patients was found. Conventional water treatment methods, such as chlorination and ozone treatments, have limited effectiveness in eliminating *T. gondii* oocysts, as demonstrated by previous research [69,70]. While drinking water has been identified as a potential source of toxoplasmosis outbreaks in various countries, including Canada [71] and Panama [72], modern municipal water treatment systems, which incorporate filtering, coagulation, flocculation, and settling processes, are generally successful in removing these oocysts [69,73].

No significant difference was observed in *T. gondii* infection rates among psychiatric patients who consumed both cooked and undercooked meat. Our findings are in line with those published by Alvarado-Esquivel et al. [74], who demonstrated a higher occurrence of *T. gondii* antibodies in patients with psychiatric disorders related to substance abuse who consumed opossum meat but not boar meat, pigeon meat, duck meat, armadillo meat, or iguana meat. The results are in contrast with those obtained by Cong et al. [58] in eastern China, who reported a significant association between *T. gondii* seropositivity and the consumption of raw/undercooked meat in psychiatric patients. This lack of difference in seroprevalence may be attributed to the low likelihood of contracting the parasite through meat consumption. Interestingly, a study conducted by Guo et al. estimated that the chance of infection from eating lamb meat is only 1.5 cases per 100,000 servings [75].

Similar to our findings, no association was noted between the consumption of undercooked meat and *T. gondii* infection in patients suffering from mental and behavioural disorders due to psychoactive substance use [74].

This research has not established a connection between *T. gondii* infection and the consumption of unwashed fruits or vegetables. In a recent study conducted in China, Lass et al., using polymerase chain reaction methods to assess vegetable samples for *T. gondii* oocysts, detected the parasite in only 3.6% of the vegetables [76], demonstrating a potentially low likelihood of infection.

In the current study, we did not find any connection between *T. gondii* infection and the consumption of alcoholic beverages, such as beer, wine, or hard spirits like liquor and brandy. A previous study conducted by Alvarado-Esquivel et al. on patients with heart disease found a potential association between alcohol consumption and *T. gondii* infection. Moreover, an association between *T. gondii* infection and alcohol was reported in individuals exhibiting risk-taking behaviour [26]. Alvarado-Esquivel et al. reported in 2021 that suicidal behaviour increased in Mexican patients attending primary healthcare if they had a history of alcohol consumption [77].

No notable difference between the number of individuals residing in the same household and the prevalence of *T. gondii* seropositivity was found. Our results align with those presented by Jones et al. [5] in their examination of the United States population, where they similarly observed no discernible association between chronic infection and household overcrowding [5].

This study has several limitations. First is the small number of study participants (<10 patients) diagnosed with certain diseases, such as adjustment disorders (F43.2), intellectual disabilities (F78.8), and somatic symptom disorders (F45.1). Second, cross-sectional studies excel at identifying associations but are unable to establish a temporal relationship, which complicates the determination of whether exposure preceded the observed outcome. Third is the participant’s recall accuracy, which can introduce measurement error. Fourth, cross-sectional studies, due to their single-time-point nature, are limited in their ability to establish causation or sequence events and primarily serve to reveal associations [78].

## 5. Conclusions

In this study, we found a high prevalence of *T. gondii* infection among psychiatric patients attending the Psychiatric Department in Arad, Western Romania. Associations were noted between *T. gondii* seropositivity and basic demographic factors like age, female gender, and residence in rural areas. Our investigation has also unveiled associations between *T. gondii* infection and certain risk factors like contact with soil, low-income levels, and limited educational attainment. Factors such as contact with pets (cats and/or dogs), hand sanitizing prior to food preparation, consumption of publicly treated water, consumption of meat and uncooked meat, unwashed horticultural products, and various types of alcohol were not found to be associated with *T. gondii* seropositivity. This study provides new information on the risk factors associated with *T. gondii* infection among psychiatric patients in Western Romania, serving as a foundation for future research and the development of preventive strategies. Further investigations should adopt longitudinal approaches to determine temporal relationships and enhance our comprehension of the intricate dynamics governing *T. gondii* transmission within this specific context. Knowledge of the risk factors associated with *T. gondii* infection could serve as a starting point for future prevention programs for psychiatric patients from Western Romania.

## Figures and Tables

**Table 1:** Prevalence of *T. gondii* IgG antibodies in psychiatric patients from Arad County, Western Romania.

Variable	Number of Investigated Patients	Number of Patients with Detectable *T. gondii* Antibodies (%)	OR	95% CI	*p* Value
Age (years)					
19–29	24	9 (37.5)	1 (Ref. *)		-
30–39	53	31 (58.49)	2.35	0.87–6.32	0.14
40–49	96	69 (71.88)	4.26	1.67–10.89	0.003
50–59	164	121 (73.78)	4.69	1.91–11.49	0.007
60–69	96	69 (71.88)	4.26	1.69–10.89	0.003
70+	31	26 (83.87)	8.67	2.45–30.69	<0.001
Area of residence					
Rural	258	191 (74.03)	1 (Ref. *)		-
Urban	206	134 (65.05)	1.54	1.03–2.28	0.04
Sex					
Female	245	182 (74.29)	1 (Ref. *)		-
Male	219	143 (65.3)	1.53	1.03–2.28	0.04
Total	464	325 (70.04)			

* Ref. = reference.

**Table 2:** Seroprevalence of *T. gondii* IgG antibodies stratified by psychiatric disease in 464 patients from Western Romania.

Mental Disorder	ICD-10 Code	Number of Investigated Patients	Number of Patients with Detectable *T. gondii* Antibodies (%)
Adjustment Disorder	F43.2	5	1 (20)
Bipolar Disorder	F31.9	27	17 (62.96)
Delusional Disorder	F05.8	27	23 (85.19)
Dementia	F03.90	29	24 (82.76)
Depression	F33.0	115	81 (70.43)
ICD *	F63.9	13	7 (53.85)
Alcohol Abuse	F10.1	14	11 (78.57)
Intellectual Disabilities	F78.8	7	6 (85.71)
Mood Disorder	F06.3	46	27 (58.7)
Other Personality and Behavioural Disorders	F07.8	72	51 (70.83)
Schizophrenia	F20.9	106	75 (70.75)
SSD **	F45.1	3	2 (66.67)
Total	-	464	325 (70.04)

* ICD = Impulse control disorder. ** SSD = Somatic symptom disorder.

**Table 3:** Prevalence of *T. gondii* IgG antibodies and associated risk factors in psychiatric patients from Arad County, Western Romania.

	*T. gondii* IgG Antibodies				
Risk Factor (Total)	Detectable (%)	Undetectable (%)	OR	95% CI	*p* Value
Contact with soil					
Yes (266)	197 (74.06)	69 (25.94)	1 (Ref. *)		-
No (198)	128 (64.65)	70 (35.35)	1.56	1.04–2.33	0.03
Contact with cats					
Yes (373)	257 (68.9)	116 (31.1)	1 (Ref. *)		-
No (91)	68 (74.73)	23 (25.27)	0.75	0.44–1.26	0.31
Contact with dogs					
Yes (219)	158 (72.15)	61 (27.85)	1 (Ref. *)		-
No (245)	167 (68.16)	78 (31.84)	1.21	0.81–1.80	0.4
Income level					
High (158)	100 (63.29)	58 (36.71)	1 (Ref. *)		-
Low (306)	225 (73.53)	81 (26.47)	0.62	0.41–0.93	0.02
Education Attainment					
Higher education (175)	110 (62.86)	65 (37.14)	1 (Ref. *)		-
Lower education (289)	215 (74.39)	74 (25.61)	0.58	0.39–0.87	0.009
Hand sanitizing prior to food preparation					
Yes (337)	235 (69.73)	102 (30.27)	1 (Ref. *)		-
No (127)	90 (70.87)	37 (29.13)	0.95	0.61–1.48	0.91
Treated public water consumption					
Yes (140)	103 (73.57)	37 (26.43)	1 (Ref. *)		-
No (324)	222 (68.52)	102 (31.48)	1.28	0.82–1.99	0.32
Meat consumption					
Yes (442)	312 (70.59)	130 (29.41)	1 (Ref. *)		-
No (22)	13 (59.09)	9 (40.91)	1.66	0.69–3.98	0.2
Uncooked meat consumption					
Yes (132)	98 (74.24)	34 (25.76)	1 (Ref. *)		-
No (332)	227 (68.37)	105 (31.63)	1.33	0.85–2.1	0.22
Unwashed horticultural product consumption					
Yes (125)	87 (69.6)	38 (30.4)	1 (Ref. *)		-
No (339)	238 (70.21)	101 (29.79)	0.97	0.62–1.52	0.91
Alcohol consumption					
Yes (303)	213 (70.3)	90 (29.7)	1 (Ref. *)		-
No (161)	112 (69.57)	49 (30.43)	1.04	0.68–1.57	0.92
Residents in the same household					
<2 residents (259)	178 (68.73)	81 (31.27)	1 (Ref. *)		-
>2 residents (205)	147 (71.71)	58 (28.29)	0.87	0.58–1.29	0.5

* Ref. = Reference.

## Data Availability

Data are contained within the article.
